# Perinatal Exposure to Environmental Endocrine Disruptors in the Emergence of Neurodevelopmental Psychiatric Diseases: A Systematic Review

**DOI:** 10.3390/ijerph16081318

**Published:** 2019-04-12

**Authors:** Fabrice Rivollier, Marie-Odile Krebs, Oussama Kebir

**Affiliations:** 1 Institut National de la Santé Et de la Recherche Médicale (INSERM), Physiopathologie des Maladies Psychiatriques, Inserm 1266 Institut de Psychiatrie et Neurosciences de Paris (IPNP), 75014 Paris, France; mo.krebs@ghu-paris.fr (M.-O.K.); o.kebir@ghu-paris.fr (O.K.); 2 Centre National de la Recherche Scientifique (CNRS), Institut de Psychiatrie, GDR 3557, 75014 Paris, France; 3 GHU Paris, Sainte-Anne, Service Hospitalo-Universitaire, 75014 Paris, France; 4 Université Paris Descartes, Sorbonne Paris Cité, Faculté de Médecine Paris Descartes, 75006 Paris, France; 5 GHU Paris, Sainte-Anne, Service d’Addictologie «Moreau de Tours», 75014 Paris, France

**Keywords:** endocrine disruptors, neurodevelopmental disorders, environmental exposure, prenatal exposure, autism spectrum disorder

## Abstract

*Background:* Exposure to endocrine disruptors is on the rise, with new compounds regularly incriminated. In animals and humans, this exposure during critical developmental windows has been associated with various developmental abnormalities, including the emergence of psychiatric disorders. We aimed to review the association between perinatal endocrine disruptor exposure and neurodevelopmental disorders in humans, focusing on cognitive and psychiatric disorders. *Methods:* We performed a systematic review with key words referring to the fields of neurodevelopment and endocrine disruptors. We reviewed 896 titles, choosing studies on the basis of titles and abstracts. We searched through the methodology sections to find perinatal exposure and neurodevelopmental disorders, following the categories indicated in the Diagnostic and Statistic Manual of Mental Disorders (5^th^ edition). References in some studies brought us to a total of 47 studies included here. *Results:* Convergent studies report an association between exposure to endocrine disruptors and autism spectrum disorder, attention-deficit hyperactivity disorder, global developmental delay, intellectual disability, communication disorders and unspecified neurodevelopmental disorders. *Conclusion:* Sufficient data exist to report that exposure to some endocrine disruptors is a risk factor for the emergence of neurodevelopmental disorders. Studying endocrine disruptor exposure in humans is still associated with some limits that are difficult to overcome.

## 1. Background

The notion of endocrine disruptor compounds (EDCs) was first used at the Wingspread Conference Center in 1994 [1]. It describes any exogenous chemical compound able to interfere with the endocrine system, resulting in adverse effects on the health of an individual and/or his/her offspring. They may not only be medicines, but also pesticides, compounds used in the plastic industry, mass consumption goods, industrial products or pollutants. Since the early nineties, some studies have reported permanent effects of EDCs on development. In 2012, the Global Assessment of the State-of-Science of Endocrine Disruptors issued by the World Health Organization and the United Nation Environment Program concluded that “different EDCs can act together and result in an increased risk of adverse effects on human health and wildlife.”

Several developmental windows are critical in terms of exposure to EDCs, especially fetal development, early childhood and puberty. In adults, exposure to EDCs can result in an immediate effect that will disappear when the exposure is gone. In children though, the exposure can cause permanent effects [2], which may appear even years later. During fetal development, it appears that environmental EDCs are able to cross the placental barrier and reach the brain [3].

A large amount of work has been done trying to investigate the effects of exposure to EDCs on global development. Evidence for reduced birth weight was reported with phthalates [4], bisphenol A (BPA) [5] and polybrominated diphenyl ethers (PBDE) [6]. Studies found a relationship between persistent organic pollutants (POPs) and male genital defects [7]. A relationship was shown between parental exposure to pesticides and increased risk of hypospadias and cryptorchidism in children [8]. Some studies found a relationship between POPs and precocious puberty or earlier menarche in girls, others found delayed puberty in boys [9]. Lastly, some links were suggested between EDCs exposure and obesity; for example, cord blood polychroeinated biphenyl (PCB) and dichlorodiphenyldichloroethylene (DDE) concentrations were associated with increased body-mass index (BMI) in 1- to 3-year-old children [10].

The endocrine system has a key role in determining how an organism adapts to its environment. Classically, EDCs act by modifying actions of endogenous hormones, stimulating or inhibiting their production, changing the way they travel through the body or altering their functions. Recent studies pointed to the fact that these mechanisms are actually very broad. Not only do EDCs act via nuclear receptor signaling, but they can also act through membrane receptors, co-activators, cell signaling and trafficking [11] as well as alterations of epigenetic programming [12].

Receptors for steroid hormones are expressed in the developing brain, playing a role in neural cell migration, differentiation synaptogenesis and myelination. Thus, they can affect behavior in a gender-specific manner in vertebrates [13]. EDCs may alter these processes by affecting those hormones/receptor complexes [14]. For example, animal studies found that prenatal/perinatal exposure to EDCs, such as PCBs, changes the volume of sexually dimorphic hypothalamic regions and affects neuronal types [15]. In zebrafish, recent results supported a disruption by ethynilestradiol of neurogenesis in the brain [16].

EDCs distribute throughout the whole body, brain included [17]. A lot of work has been done lately trying to find an association between neurodevelopment and EDCs exposure. Animal models have suggested relations between EDCs exposure and behavior abnormalities [18], spatial difficulties [19], hyperactivity [20] and anxiety [21]. Animal models hardly match the complexity of psychiatric diseases in humans. Moreover, in the literature, we did not find any cohesive reviews adopting a clinician point of view. Indeed, most studies focus on one EDC, and describe its potential neurodevelopmental effects. Here, we aim to review the state of the art on environmental EDCs exposure in relation to neurodevelopmental cognitive and psychiatric disorders in humans. We chose a cohesive perspective, tailored for clinicians, presenting our results according to already well-described psychiatric disorders.

## 2. Materials and Methods

Our eligibility criteria did not screen participants on age. Children and adults were both included since the neurodevelopmental window can be very large, with symptoms appearing years later. Studies followed a prospective or retrospective observational design (due to obvious ethical questions). They may, or may not, have had a control group. Indeed, some EDCs being ubiquitous in our environment, comparisons could only be assessed according to high vs. low exposure. The psychiatric assessment needed to follow a standardized method. We reviewed studies that had been published until 1 December 2018, exposing us to publication and selective reporting within studies biases. We screened the PubMed and Embase databases. Regarding exposure, we searched the terms: “endocrine disruptors,” “bisphenol,” “phthalates,” “pollutants,” “polybrominated diphenyl ethers” (PBDE), “hexaclorobenzene” (HCB), “polychlorinated biphenyls” (PCB), “metal,” “solvents,” “polycyclic aromatized hydrocarbons” (PHA), “chlorpyfiros” (CPH), and “organohalogens compounds” (OHC). Regarding the outcomes we used the terms: “neurodevelopment,” “attention deficit disorder hyperactivity” (ADHD), “autism spectrum disorder” (ASD), “intellectual disabilities” (ID), “learning disorders” (LD), “developmental delay,” “language” and “behavior”. We first reviewed titles and abstracts. We then searched for the methodology to find our eligibility criteria.

## 3. Results 

**Figure 1:**
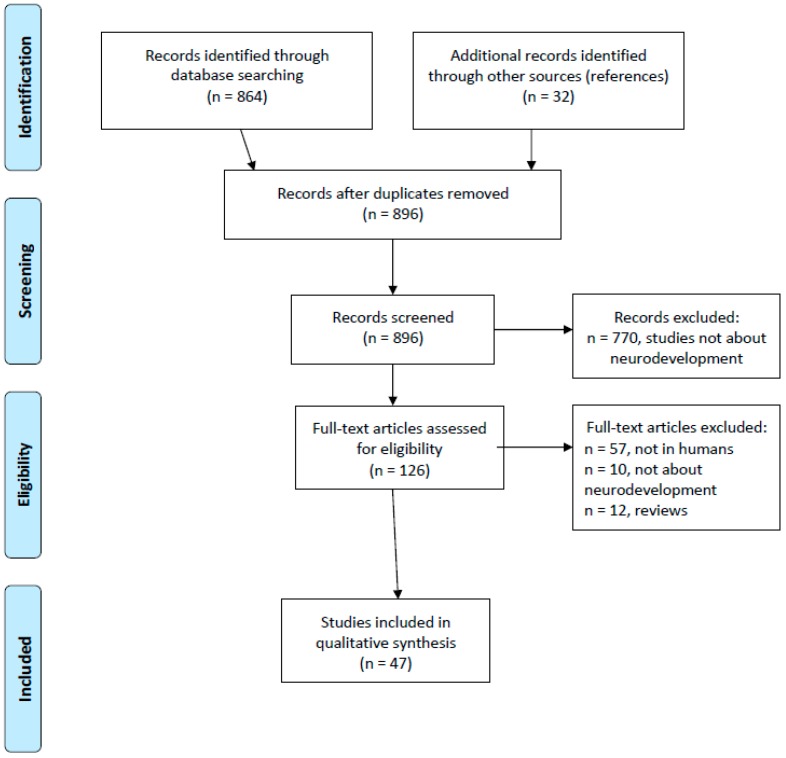
PRISMA flow diagram. Source: Moher D. et al., the PRISMA group (2009) [22].

We reviewed 864 titles and found 32 other titles through references in screened articles. We chose studies based on title and abstract. A total of 770 studies that did not concern neurodevelopment were excluded. We then carefully searched for the methodology to find human exposure, perinatal exposure and neurodevelopmental disorders assessed with a standardized method. A total of 79 studies that did not meet these criteria were excluded, which brought us to a total of 47 studies included here (PRISMA chart in Figure 1). 

The results are presented here according to neurodevelopmental categories as defined in the Diagnostic and Statistic Manuel of Mental Disorders (DSM) (ADHD, ASD, ID, global developmental delay, communication disorders and unspecified disorders).

### 3.1. EDCs Exposure and ASD 

ASD is characterized by persistent deficits in social communication and social interaction across multiple contexts, including deficits in social reciprocity, nonverbal communicative behaviors used for social interaction, and skills in developing, maintaining, and understanding relationships. In addition to social communication deficits, the diagnosis of ASD requires the restricted or repetitive patterns of behavior, interests, or activities. There has been a large increase in the diagnosis of ASD in recent decades [23]. One of the main explanations could be the use of better tools designed to diagnose the disease. As of today, the diagnosis itself is also better understood and recognized. The model of a multifactorial disease with complex genetic factors interacting with environmental factors has been advanced. Based on that idea, we can hypothesize that changes in the environment could, at least partially, explain the recent increase in the number of cases.

The results of ten studies [24,25,26,27,28,29,30,31,32,33], published between 2006 and 2017, are shown in Table 1. ASD was assessed as a clinical category in six studies while four others investigated autistic traits through social competence evaluation. We noticed that the first six studies have very different designs, which make them difficult to compare. Windham and Nishijo both studied prenatal exposure with follow-up studies [24,25]. Nishijo’s cohort included younger children and the diagnosis was made using a scale, not through a clinical examination, unlike other studies. To these six studies, we added four more studies that found an association between EDCs exposure and autistic traits, measured using scales about social competence [26,27,28,29].

Five authors found significant associations between an ASD diagnosis and exposure to phthalates, air pollutants, 2,3,7,8-Tetrachlorodibenzodioxin (TCDD) and BPA [24,25,26,27,28]. Only Rahbar et al. did not find any association between an ASD diagnosis and postnatal exposure to different EDCs (dioxins, dibenzofurans, BPA and phthalates), though this cohort was small and a few patients were under the range of detection threshold for each EDC [33].

### 3.2. EDCs Exposure and ADHD

ADHD is a neurodevelopmental disorder defined by impaired levels of attention, disorganization, and/or hyperactivity-impulsivity. As for ASD, diagnoses of ADHD have been increasing lately. Population surveys suggest that ADHD occurs in most cultures in about 5% of children and about 2.5% of adults. Changes in our environment, in association with better tools for diagnosis, could explain this observation [34].

The results of thirteen studies [26,27,35,36,37,38,39,40,41,42,43,44,45], published between 2003 and 2016, are shown in Table 2. Jacobson et al., in 2003, first showed a correlation between “ADHD-like” profiles and prenatal exposure to PCB [35]. Seven years later, Sagiv found a similar result [36]. 

Three studies using the same cohort from Minorca (Spain) found an association between the diagnosis and PBDE, HCB and nitrogen dioxide [26,27,37]. Another study found an association with PBDE exposure (mostly postnatal) [38].

Regarding BPA, prenatal exposure was not found to be associated with ADHD [37], although postnatally, the results seem to differ according to two studies [39,40].

Once again, one main difference among all studies is the way ADHD is diagnosed. In some, the outcome is an ADHD diagnosis, as defined in the DSM, while in others, the outcome is defined as “ADHD-traits.” It has been suggested that ADHD psychopathology can be viewed dimensionally, with symptoms distributed continuously in the general population [46,47]. Thus, healthy individuals may present high and low levels of ADHD-traits, altering their functioning, but not display enough symptoms to suffer from an ADHD, in the clinical sense. 

### 3.3. EDCs Exposure and Global Developmental Delay (GDD)

Global developmental delay is diagnosed when an individual fails to meet expected milestones in several areas of intellectual functioning. The diagnosis is made with systematic assessment of intellectual functioning such as the Bayley Scale of Infant Development (BSID) or the Gesell Developmental Schedules (GDS). These tests are chosen according to the individual’s age. The results of seven studies, published between 2006 and 2012, are shown in Table 3 [48,49,50,51,52,53,54]. Most of these studies present a positive association between prenatal exposure to EDCs and a decrease in mental and/or psychomotor index. Two studies found an association with phthalates [48,51], and two studies also found an association with polycyclic aromatic hydrocarbon (PAH) [50,53]. Noteworthy, Perera et al. presented recent results in favor of a reversible decrease of developmental quotient after a source of PAH was removed [54].

### 3.4. EDCs Exposure and Intellectual Disability

Intellectual disability is characterized by deficits in general mental abilities. The deficit results in the impairment of adaptive functioning in the conceptual, social and practical domains. It is typically measured using psychometry valid tests. Those tests need to be adapted to the child’s age. The results of five studies, published between 2009 and 2017, are shown in Table 4 [55,56,57,58,59]. 

Two studies from two different cohorts found convergent results in favor of an association between exposure to PAH and intellectual quotient (IQ) alterations. We notice that they used two different methods to assess the IQ, and in one study [57], the exposure was 10 times higher than in the other [58]. Two studies searching for an association with PCB exposure reported inconsistent results, but again, the exposure itself and PCB concentrations differed [55,56].

### 3.5. Exposure to EDCs and Communication Disorder

Disorders of communication include deficits in language, speech and communication. They are assessed with standardized measures that must take into account the individual’s cultural and linguistic context. Till et al. found a significant association between high prenatal exposure to organic solvents and difficulties in receptive (*p* = 0.25) and expressive (*p* = 0.44) language as well as graphomotor abilities (*p* = 0.04) when compared to low exposure [60]. The results are summarized in Table 5.

### 3.6. ED Exposure and Behavior/Unspecified Disorders

Here, we included symptoms (mostly behavioral symptoms) that cause impairment in social, occupational, or other important areas of functioning, that do not meet the full criteria for any of the disorders in the neurodevelopmental disorders diagnostic class (“unspecified neurodevelopmental disorders” as defined in DSM-5). 

The results of thirteen studies published between 1994 and 2017 are shown in Table 6. Six studied perinatal exposure to BPA. Among these, three studies used the Health Outcome and Measures of the Environment (HOME) cohort and showed no associations between behavior and BPA exposure at 5 weeks [61], but found an association in girls, aged 2 and 3 years old [62,63]. In older children (aged 10 years old), three other studies found an association between urinary BPA concentration and behavior [64,65,66].

Five studies examined the consequence of prenatal exposure to phthalates. At 5 days, no association was found [67]. At 5 weeks, authors found an association with decreased regulation, decreased handling, and nonoptimal reflexes [61]. In older children (3–9 years old), all three studies found an association with several behavioral abnormalities [68,69,70]. Finally, two studies found an association with prenatal exposure to PCB and behavior [71,72].

## 4. Discussion

The aim of this review was to present an overview of the known effects of EDCs on the emergence of cognitive and psychiatric neurodevelopmental disorders. We chose clinical approach and presented our results with categories based on DSM classification, aware that it may not corroborate the pathophysiological processes underlying each disease. We believe this different approach may be useful to clinicians, presenting our results in a more clinical and cohesive manner and providing them guidance in going through patients’ medical histories. Numerous recent studies have shown that exposure to EDC can be linked to specific or less specific neurodevelopmental disorders. However, a number of issues make it difficult to characterize the relation between the exposure and the outcome.

- First, the exposure itself can be difficult to define in terms of duration and moment of exposure. The specific measurement of one exposure may be difficult to obtain. As of today, more than 800 products are considered as EDCs, some being commonly found in our environment. We are probably mostly studying mixture effects, and it may be difficult to isolate one specific exposure [74]. Long-lived EDCs are easier to study than many other EDCs (such as BPA and phthalates) that are quickly metabolized in humans and rapidly degraded in the environment. Some papers have tried to overcome this issue in animals, but it has yet to be done in humans [75].
  In humans, evidence is mostly based on correlations between concentrations of chemicals and outcomes, assessed at one point during an individual’s life, which is not a real-world situation. At some very specific times, even in very low concentrations, any exogenous EDCs may exceed the body’s natural endogenous hormone levels, changing target cells that are sensitive to hormones. Thus, even extremely low dosages of EDCs can alter biological outcomes. Moreover, studies showed that, due to nonmonotonic dose–response curves, the effects of low doses cannot be predicted by the effects observed at high doses [76].
- Second, the pathophysiological processes involved with EDCs have not been clearly elucidated yet. If, by definition, they are all able to alter the physiological endocrine system, their pathophysiology may differ or overlap. EDCs interfere with the endocrine system in multiple ways, making it difficult to link one pathway to one symptom. They can act directly on the behavior and development via sexual hormone perturbations (for example, the anti-androgenic effect of phthalates [77]), but they could interfere directly with the neuronal development as well; for instance, interactions with the vasopressin system [78] and oxytocin [79] have been described, among others.
- Third, the outcome measurement is most often based on neuropsychological assets that may not correlate to a clinical diagnosis, but more to a ‘profile.’ The fact that diagnosis in the developmental disorder categories co-occur frequently makes it difficult to determine the specificity of the cause–effect relationship [80].

Under the ‘neurodevelopmental disorders’ section, the DSM-5 includes disorders such as intellectual disabilities, communication disorders, ASD, ADHD, specific learning disorders and motor disorders. Those disorders are mainly diagnosed in children. We know now that other psychiatric disorders, such as schizophrenia, diagnosed years later, are also considered as neurodevelopmental disorders. We could assume that perinatal exposure to EDCs also has an impact in these ‘adult’ disorders [81]. Although research in the field is still scarce [82], an effort in that direction now seems crucial.

Finally, the expanding work around EDCs is recently taking a new turn with the notion of epigenetic transgenerational inheritance and of all its possible consequences [82]. Indeed, it has been reported that prenatal exposure to an EDC may not only cause adult late-onset disease, but may affect future generations through the germline [83]. Transgenerational behavioral phenotypes of development exposure have indeed been shown for two EDCs, namely vinclozin [84] and BPA [85]. These transgenerational effects may be supported by molecular alterations to the germline, promoting effects on subsequent generations. As reported in a recent communication by Skinner, recent work suggests that endocrine disruptors may alter the epigenetic programming of the germline, these changes being transmitted across generations in the absence of direct exposure [86]. 

## 5. Conclusions

Sufficient data exist to report that exposure to some EDCs participates in the emergence of neurodevelopmental disorders. This may account for the growing incidence of neurodevelopmental disorders observed in the general population. Despite the current effort in the field, the specificity of this link in terms of exposure and outcome will be difficult to assess. Indeed, the measurements of exposure, use of biomarkers and neuropsychological tools used for outcome all show an extreme variability in the literature, making it difficult to accumulate evidence and identify pathophysiological processes. While EDCs are increasingly prevalent in our environment, more cohorts in humans and experimental studies in animals are urgently needed to bring a more comprehensive picture of the long-term neurodevelopmental consequences of EDCs, as well as to define the precise windows of vulnerability. 

## Figures and Tables

**Table 1:** Endocrine disruptors and autism spectrum disorders.

Authors	Outcome	Exposure	Dose (Medians)	Population	Results
Testa et al., 2012 [32]	ASD diagnosis (DSM-IV)	Phthalates (postnatal)	Urinary samples 5-OH-mono-ethylhexin phthalates (MEHP) = 0.18 µg/L 5-oxo-MEHP = 0.096 µg/L	n = 48 ASD (mean 11 years) + 45 controls	Higher rates of 5-OH-MEHP, 5-oxo-MEHP and MEHP in ASD patients (*p* < 0.05).
Windham et al., 2006 [24]	ASD diagnosis (DSM-IV)	Air pollutants (prenatal)	Estimated concentrations by US Environmental Protection Agency (EPA)	n = 284 ASD (mean 12 years) + 657 controls	Ajusted odds-ratio (AORs) elevated by 50% in top quartile of chlorinated solvents and heavy metals (IC 1.1–2.1).
Volk et al., 2011 [30]	ASD diagnosis (clinical diagnosis)	Air pollutants (neonatal)	Distance to freeways	n = 304 ASD (5–14 years) + 259 controls	Association of maternal residence at birth near a freeway (≤ 309 m) with ASD odds-ratio (OR) = 1.86 (1.04–3.45).
Rahbar et al., 2017 [33]	ASD diagnosis (DSM-5)	BPA, phthalates, dioxins and dibenzofurans (postnatal)	Serum samples (dioxins and dibenzofurans) and urine samples (BPA and phthalates)	n = 30 ASD (2–8 years) + 10 controls	Mean concentrations did not differ between the ASD cases and control group (*p* ≥ 0.27).
Nishijo et al., 2014 [25]	ASD diagnosis (Autism Spectrum Rating Scale (ASRS) and Bailey III)	TCDD (pre and postnatal)	Milk sample	n = 153 (3 years)	The high TCDD groups showed higher ASRS scores than the mild-TCDD groups (*p* = 0.042). No differences in neurodevelopmental scores.
Stein et al., 2015 [31]	ASD diagnosis (DSM-IV-TR)	BPA (postnatal)	Urine sample	n = 46 ASD (mean 10 years) + 52 controls	Association between ASD and total BPA and bound BPA (*p* < 0.05).
Ribas et al., 2007 [26]	Social competence California preschool social competence scale (CP-SCS))	HCB (prenatal)	Cord serum HCB = 0.73 ng/mL	n = 477 (4 years)	Association of postnatal exposure (HCB > 1.5 ng/mL) with social impairment: Relative risk (RR) 4.04 (1.76–9.58).
Gascon et al., 2011 [27]	Social competence (CP-SCS)	PBDE (pre and postnatal)	Cord blood PBDE 47 at birth = 2.10 ng/g lipid at 4 years = 0.12 ng/g lipid	n = 422 (4 years)	Association of postnatal exposure with social impairment: RR = 2.6 (1.2–5.9).
Miodovnik et al., 2011 [28]	Social competence (Social Responsiveness Scale (SRS))	Phthalates and BPA (prenatal)	Urinary samples BPA 1.25 µg/L LMW phthalates 419 µg/L	n = 137 (7–9 years)	Association of LMWP with social impairment *β* = 1.53 (*p* < 0.05). No association with BPA.
Nowack et al., 2015 [29]	Autistic Traits (SRS)	Polychlorinated dibenzodioxins (PCDD) and PCB (prenatal)	Maternal blood samples PCDD = 12.91 pg/g PCB = 6.85 mg/g	n = 100 (10 years)	Negative associations between PCDD levels and SRS scores in the whole group (*p* < 0.05) in girls and in one subscale (social motivation) in boys. For PCB, associations with one subscale for the whole group (autistic mannerisms).

**Table 2:** Endocrine disruptors and attention-deficit hyperactivity disorder.

Authors	Outcome	Exposure	Dose (Medians)	Population	Results
Gascon et al., 2011 (2nd part) [27]	ADHD (DSM-IV Criteria, Connors Scale)	PBDE (pre and postnatal)	PBDE 47 Cord blood (birth) = 2.10 ng/g Blood sample (4 years) = 0.12 ng/g	n = 422 (4 years)	Association of postnatal exposure with ADD RR = 1.8 (1.0–3.2). No prenatal association.
Ribas et al., 2009 (2nd part) [26]	ADHD (DSM-IV Criteria, Connors Scale)	HCB (prenatal)	Cord serum = 0.73 ng/mL	n = 477 (4 years)	When HCB > 1.5 ng/mL, association of prenatal exposure with ADHD, RR= 2.71 (1.05–6.96).
Sagiv et al., 2009 [36]	ADHD (DSM-IV Criteria, Connors Scale)	PCB and p,p′-dichlorodiphenyldichloroethylene (DDE) (prenatal)	Cord serum = 0.19 ng/g lipid	n = 573 (mean 8.2 years)	Association of ADHD Index/DSM-IV criteria with PCB and p,p′-DDE levels, *p* < 0.05.
Eskenazi et al., 2013 [38]	ADHD (DSM-IV Criteria, Connors Scale)	PBDE (pre and postnatal)	Maternal serum PBDE10 = 28.7 ng/g Child serum (7 years) PBDE10 = 90.9 ng/g	n = 310 (5 years) + 323 (7 years)	Association of prenatal exposure with Connors ADHD DSM-IV total scale AOR = 2.6 (0.2–5.0) in 5-year-old children. Association of postnatal exposure with hyperactivity AOR = 4.8 (0.5–9) and inattention AOR = 2.9 (0.4–5.5) on BASC2 teacher report in 7-year-old children.
Morales et al., 2009 [37]	ADHD (DSM-IV Criteria Connors Scale)	Gas appliance and nitrogen dioxide (neonatal)	NO2 concentration = 15.8 ppb	n = 482 (4 years)	Association of use of gas appliances with ADHD symptoms OR = 2.72 (1.01–7.28). Association of nitrogen dioxide concentrations with ADHD symptoms, OR = 1.04 (1.00–1.09).
Arbuckle et al., 2016 [40]	ADHD & Learning (Strenght and difficulties questionnaire (SDQ))	Phthalates, BPA and lead (postnatal)	Blood sample (lead) and urine sample (BPA and phthalates)	LD n = 94, ADD/ADHD n = 49, (6–11 years)	Association of lead exposure with ADHD (*p* = 0.047).
Janulewicz et al., 2008 [41]	ADHD diagnosis (clinical)	PCE (pre and postnatal)	Estimation in water by area Prenatal = 7.34 g Postnatal = 20.34 g	n = 1063 exposed vs. n = 1023 unexposed (5 years)	No association with ADHD.
Harley et al., 2013 [39]	Behavior ((Behavior assessment system forchildren (BASC2) & Conners Scale)	BPA (pre and postnatal)	Urinary maternal (birth) = 1.1 µg/L Child (5 years) = 2.5 µg/L (geometric mean)	n = 292 (7 years)	No association of prenatal exposure with ADHD. Association of postnatal exposure with ADHD in girls on mothers β = 1.3 (0.2–2.3) and teachers report β = 1.7 (0.3–3.1). Association with inattention in boys in teachers report β = 1.7 (0.3–3).
Laslo et al., 2004 [42]	Neuropsychological Profile (with Connors Rating Scale)	Organic solvents (prenatal)	Interrogation	n = 32 (3–7 years)	Association with ADHD β = 0.62 (*p* < 0.02). No association with IQ.
Chopra et al., 2014 [43]	ADHD & LD (diagnosis)	Phthalates (postnatal)	Urine sample	ADD n = 102, LD n = 173, both n = 56 (6–15 years)	Association of ADD with urinary concentration of di–2-ethylhexyl phthalates OR = 2.1 (1.1–3.9) and phthalates OR = 2.7 (1.2–6.1). No association with LD.
Verner et al., 2010 [44]	Behavior (Behavior rating scale (BRS) & BSID-II)	PCB (pre and postnatal)	Cord blood PCB-153 = 112.3 ng/g lipid	n = 168 (11 months)	Correlation of prenatal PCB-153 level with inattention r 0.205 (*p* < 0.05) and of postnatal levels with hyperactivity r 0.181 (*p* < 0.05).
Perera et al., 2011 [45]	Behavior (Child behavior checklist (CBCL))	PAH (prenatal)	Cord blood 32P = 2.45 adducts/10^8^ nt	n = 215 (followed 8 years)	Association of exposure and attention problems at 4,8 years β = 0.38 (0.06–0.69) and 7 years β = 0.22 (0.06–0.38)
Jacobson et al., 2003 [35]	ADHD like neuropsychological profile (continuous performance test (CPT), DigitCancellation, etc.)	PCB (prenatal)	Cord serum PCB = 2.7 ng/mL	n = 144 (11 years)	Correlation between exposure and attention deficit r = 0.17 and working memory r= 0.22 (*p* < 0.05)

**Table 3:** Endocrine disruptors and global developmental delay.

Authors	Outcome	Exposure	Dose (Medians)	Population	Results
Kim et al., 2011 [48]	Development (BSID-II)	Phthalates (prenatal exposure)	Urinary mono (2-ethyl-5-hydroxyhexyl) phthalates (MEHHP) = 8.9 μg/L (mean)	n = 460 (6 months)	Association of mental development index (MDI) with MEHHP β = −0.97 (−1.85 −0.08) and mono (2-ethyl-5-oxohexyl) phthalates MEOHP β = −0.95 (−1.87 to −0.03). Association of PDI with MEHHP β = −1.20 (−2.33 to −0.08).
Herbstman et al., 2010 [49]	Development (BSID-II) and Intelligence (Wechsler preschool and primary scale of intelligence (WPPSI-R))	BPDE (prenatal exposure)	Cord blood PBDE47 = 11.2 ng/g lipid PBDE99 = 3.2 ng/g lipid PBDE100 = 1.4 ng/g lipid	n = 96 (3 years)	Association of 24-month MDI (BDE-47, 99, and 100), 48-month full-scale β = −3.29 (−5.95 −0.63) and performance intellectual quotient (IQ) (Brominated diarylethers (BDE)-100).
Perera et al., 2006 [50]	Development (BSID-II/CBCL)	PAH (prenatal exposure)	Air concentrations measures	n = 181 (followed 3 years)	No association of prenatal exposure to PAHs with psychomotor developmental index (PDI) or behavioral problems. Association of high prenatal exposure to PAHs (upper quartile) with lower MDI at age 3 β = −5.69 (−9.05 to −2.33)
Whyatt et al., 2012 [51]	Development (BSID-II/CBCL)	Phthalates (prenatal exposure)	Urinary MiBP = 9.3 μg/L MnBP 38.0 μg/L (mean)	n = 319 (3 years)	Association of PDI scores with mono-n-butyl phthalates MnBP β = −2.81 95% (−4.63, −1.0) and monoisubitil phthalates (MiBP) β = −2.28 (−3.90, −0.67). In girls, association of MDI scores with MnBP β = −2.67 (−4.70, −0.65).
Rauh et al., 2006 [52]	Development (BSID-II/CBCL)	CPF (prenatal exposure)	Umbilical cord blood	n = 234 (3 years)	Association of highly exposure (>6.17 pg/g plasma) with PDI at 3 years compared with lower levels (*p* = 0.006). Association of highly exposure with ADHD at 3 years of age compared with those with lower levels of exposure (*p* = 0.018).
Tang et al., 2008 [53]	Development (GDS)	PAH (prenatal exposure)	Cord blood adducts = 0.32 adducts/10^8^ nt (mean)	n = 110 (followed 2 years)	Association of increased adduct levels with decreased average GDS developmental quotient (DQ) β = −14.58 (−28.77 to −0.37).
Perera et al., 2008 [54]	Development (GDS)	PAH (prenatal exposure)	Cord blood adducts = 0.20 adducts/10^8^ nt (mean)	n = 107 (followed 2 years)	No association of adduct levels with average GDS DQ.

**Table 4:** Endocrine disruptors and intellectual disability.

Authors	Outcome	Exposure	Dose (Medians)	Population	Results
Cho et al., 2010 [59]	Intelligence (K-Wechsler intelligence scale for children (WISC))	Phthalates (postnatal)	Urinary MEHP = 21.3 μg/L, MEOHP = 18.0 μg/L and MPB = 48.9 μg/L (geometric mean)	n = 667 (9 years)	Association of full-scale IQ and verbal IQ scores with MEHP; β = −1.93 and β = −0.91 (*p* < 0.05) and MEOHP metabolites β = −0.91 and β = −0.81 (*p* < 0.01) but not with MBP metabolites.
Edwards et al., 2010 [57]	Intelligence (Raven’s colored progressive matrices (RCPM))	PAH (prenatal)	PAH in air = 39.62 ng/m^3^ (mean)	n = 214 (5 years)	Association of higher (>17.96 ng/m^3^) prenatal exposure to airborne PAHs with decreased RCPM scores at 5 years of age β = −1.36 (−2.48 −0.23).
Perera et al., 2009 [58]	Intelligence (WPPSI-R)	PAH (prenatal)	PAH in air = 3.48 ng/m^3^ (mean)	n = 249 (5 years)	Association of high PAH levels (>2.26 ng/m^3^) with full-scale IQ β = −4.307 (*p* = 0.007) and verbal IQ β = −4.668 (*p* = 0.003).
Zhang et al., 2017 [56]	Reading (Woodcock-Johnson (WJ)-III and Wide range achievement test (WRAT)-4), intelligence (WISC-IV) and Behavior (BASC-2)	PBDE and PCB (prenatal)	Maternal serum (Sum4PBDEs = 35.65 ng/g) (Sum4PCBs = 31.30 ng/g)	n = 239 (at 5 and 8 years)	Association of Sum4PBDE with reading composite score and FISQ, and externalizing problems at 8 years (not at 5). No association with Sum4PCB
Lai et al., 2002 [55]	Behavior (WPPSI-R, CBCL and Rutter’s)	PCBs (prenatal)	Interrogation (contaminated oil)	n = 118 (followed 4 years) + 118 controls	Exposed children scored 3 points lower than controls for IQ (*p* < 0.05, 3 points higher on the CBCL (*p* = 0.002) and 6 points higher (*p* = 0.001) on the Rutter’s scale.

**Table 5:** Endocrine disruptors and communication disorders.

Authors	Outcome	Exposure	Dose	Population	Results
Till et al., 2001 [60]	Behavior (NEPSY and CPT)	Organic solvents (prenatal)	Interrogation/checklist	n = 33 (3–7 years)	Association of high exposure with difficulties in receptive (*p* = 0.025) and expressive (*p* = 0.044) language as with graphomotor abilities (*p* = 0.004) when compared to low exposure.

**Table 6:** Endocrine disruptors and unspecified neurodevelopmental disorders.

Authors	Outcome	Exposure	Dose (Medians)	Population	Results
Chen et al., 1994 [71]	Behavior (Rutter’s Child Behavior Scale and Werry Weiss Peter’s Activity Scale)	PCB (prenatal)	Interrogation (contaminated oil)	n = 115 (followed 6 years) + 115 controls	Exposed children found to have scores 7% to 43% (mean = 23%) higher than the control children on the Rutter scale at every time point.
Braun et al., 2011 [63]	Behavior (BASC2 and behavior rating inventory of executive function (BRIEF)).	BPA (pre and postnatal)	Urinary BPA = 1.2 ng/mL	n = 239 (followed 3 years)	In girls, association of prenatal exposure to BPA in with BASC-2 and BRIEF-P scores (increased 9 to 12 points). No associations of postnatal BPA exposure with behavior.
Braun et al., 2009 [62]	Behavior (BASC2)	BPA (prenatal)	Urinary BPA = 1.3 ng/mL	n = 249 (2 years)	In girls, association of BPA exposure with externalizing scores β = 6.0 (0.1–12.0).
Plusquellec et al., 2010 [72]	Behavior (BSID-II)	PCBs, Hg and Pb (pre and postnatal)	Cord blood: PCB-153 = 120.6 µg/kl Pb = 5µg/dL, Hg = 22.2 µg/L (mean). 5 years old blood: PCB-153 =159 µg/dL Pb = 5.4 µg/dL Hg = 9.6 µg/L (mean)	n = 110 (5 years)	Association of postnatal exposure to Pb with impulsivity and irritability β = 0.20 (*p* < 0.05). Association of prenatal exposure with PCB 153 with anxiety β = 0.26 (*p* < 0.01). Association of postnatal exposure with global activity latency β = −0.25 (*p* < 0.05).
Perez et al., 2015 [64]	Behavior (CBCL)	BPA (postnatal)	Urine sample = 18.48 microg/L	n = 300 (9–11 years)	Higher BPA concentrations associated with worse behavioral scores on several syndrome scores such as somatic complaints (*p* = 0.015), social problems (*p* = 0.043) and thought problems (*p* = 0.017).
Evans et al., 2014 [65]	Behavior (CBCL)	BPA (prenatal)	Urine sample at 27 weeks of pregnancy 1.10 microg/L	n = 153 (6–10 years)	We observed a significant interaction between maternal urinary BPA and sex for several behaviors (externalizing, aggression, anxiety disorder, oppositional/defiant disorder and conduct disorder traits), but no significant associations between BPA and scores on any CBCL scales.
Hong et al., 2013 [66]	Behavior (CBCL/Learning Disability Evaluation Scale (LDES))	BPA (postnatal)	Urinary BPA = 1.32 lg/g Cr (geometric mean)	n = 1089 (8–11 years)	Significant (*p* < 0.05) association between exposure and internalizing problems β = 1.07, attention problems β = 1.22, social problems β = 0.93 and anxiety/depression β = 0.66. On the LEDS, significant associations were found for thinking β = −0.36, writing β = −0.31, calculations β = −0.43 and learning quotient β = −1.90.
Yolton et al., 2011 [61]	Behavior (NICU Network Neurobehavioral Scale (NNNS))	BPA (prenatal)	Urinary BPA = 1.7 ng/mL (mean at 26 weeks)	n = 350 (5 weeks)	No association of prenatal exposure to BPA was found with neurobehavioral traits.
Yolton et al., 2011 (2nd part) [61]	Behavior (NNNS)	Phthalates (prenatal)	Urinary DBP sum = 113 μM/L DHEP sum = 245 μM/L (mean)	n = 350 (5 weeks)	Association of higher DBP metabolites with decreased regulation (*p* = 0.04), decreased handling (*p* = 0.02). Association of higher total (DEHP) metabolites with more nonoptimal reflexes (*p* = 0.03).
Swan et al., 2010 [70]	Behavior (Pediatric Attachment Style Indicator (PASI))	Phthalates (prenatal)	Urinary DBP sum in boys = 35.6 μM/L in girls = 42.5 μM/L DHEP sum in boys = 23.4 μM/L in girls = 27.0 μM/L (mean)	n = 143 (mean 60 months for boy and 59 months for girls)	Associations of MiBP and DHEP with a decreased (less masculine) composite score in boys r = −4.53 and −4.20 (*p* = 0.01 and 0.04). Associations of MEHHP and MEOHP and the DEHP sum with decreased masculine score r = −3.29, −2.94 and −3.18 (*p* = 0.02, 0.04 and 0.04), respectively.
Philippat et al., 2017 [68]	Behavior (SDQ)	Phthalates and phenols (among them is BPA) (prenatal)	Urine sample during pregnancy	n = 529 (3.1 years old) and n = 464 (5.6 years)	BPA was associated with relationship problems at 3 years and hyperactivity/inattention at 5. MnBP was associated with internalizing behavior, relationship problem and emotional symptoms at 3. MPzP was associated with internalizing problems and relationship problems at 3.
Engel et al., 2010 [69]	Behavior and Cognitive functioning (BASC and BRIEF)	Phthalates (prenatal)	Urinary low-molecular-weight phthalates LMWP = 1.88 μM/L	n = 188 children (4–9 years)	Association of LMWP exposure with conduct problems β = 2.40 (1.34–3.46)
Engel et al., 2009 [67]	Neonatal Behavior (Brazelton Neonatal Behavioral Assessment Scale BNBAS)	Phthalates (prenatal)	Urinary Sum LMW = 2.23 μM/L Sum high molecular weight (HMW) = 0.46 μM/L	n = 295 (5 days)	No associations of exposure to phthalates and BNBAS scores.
Roze et al., 2009 [73]	Neuropsychological Profile (WPSSI-R and NEPSY-II)	Organohalogens (prenatal)	Maternal serum BDE-47a = 0.9 ng/g lipid (mean)	n = 62 (5–6 years)	For specified metabolites, association of exposure to brominated flame retardants with worse attention, better coordination, better behavior and better total intelligence. Association of exposure to chlorinated OHCs with less choreiform dyskinesia, worse fine manipulative abilities, worse inhibition and worst behavior. Association of PCP with worse coordination and worse performance intelligence.
